# The Long Linker Region of Telomere-Binding Protein TRF2 Is Responsible for Interactions with Lamins

**DOI:** 10.3390/ijms22073293

**Published:** 2021-03-24

**Authors:** Aleksandra O. Travina, Nadya V. Ilicheva, Alexey G. Mittenberg, Sergey V. Shabelnikov, Anastasia V. Kotova, Olga I. Podgornaya

**Affiliations:** 1 Institute of Cytology RAS, 194064 St. Petersburg, Russia; nad9009@yandex.ru (N.V.I.); mittenberg@incras.ru (A.G.M.); sergey_shabelnikov@incras.ru (S.V.S.); anastkotova@gmail.com (A.V.K.); 2 Stem Cell Bank Pokrovsky, 199106 St. Petersburg, Russia; 3 Department of Cytology and Histology, St. Petersburg State University, 199034 St. Petersburg, Russia

**Keywords:** telomere, TRF2, chromosome organization, lamins, nuclear lamina

## Abstract

Telomere-binding factor 2 (TRF2) is part of the shelterin protein complex found at chromosome ends. Lamin A/C interacts with TRF2 and influences telomere position. TRF2 has an intrinsically disordered region between the ordered dimerization and DNA-binding domains. This domain is referred to as the long linker region of TRF2, or udTRF2. We suggest that udTRF2 might be involved in the interaction between TRF2 and lamins. The recombinant protein corresponding to the udTRF2 region along with polyclonal antibodies against this region were used in co-immunoprecipitation with purified lamina and nuclear extracts. Co-immunoprecipitation followed by Western blots and mass spectrometry indicated that udTRF2 interacts with lamins, preferably lamins A/C. The interaction did not involve any lamin-associated proteins, was not dependent on the post-translation modification of lamins, nor did it require their higher-order assembly. Besides lamins, a number of other udTRF2-interacting proteins were identified by mass spectrometry, including several heterogeneous nuclear ribonucleoproteins (hnRNP A2/B1, hnRNPA1, hnRNP A3, hnRNP K, hnRNP L, hnRNP M), splicing factors (SFPQ, NONO, SRSF1, and others), helicases (DDX5, DHX9, and Eif4a3l1), topoisomerase I, and heat shock protein 71, amongst others. Some of the identified interactors are known to be involved in telomere biology; the roles of the others remain to be investigated. Thus, the long linker region of TRF2 (udTRF2) is a regulatory domain responsible for the association between TRF2 and lamins and is involved in interactions with other proteins.

## 1. Introduction

Telomeric DNA is composed of noncoding, double-stranded, highly conserved repeat sequences and is associated with the shelterin protein complex. Human shelterin consists of six proteins: TRF1, TRF2, Rap1, TIN2, TPP1, and POT1 [1,2]. Telomeres along with their protein components are involved in the maintenance of the integrity and stability of eukaryotic genomes, the regulation of gene expression, and chromatin organization. Details of the latter function of telomeres have not yet been fully elucidated.

Telomeres co-fractionate with the nuclear matrix (NM) in NM preparations [3,4]. The concept of NM, a karyoskeletal structure supporting the genome and its activities, was popular in the 20th century and stimulated many studies. In this paper, the term NM refers to the preparation obtained using a previously described method [5,6,7], which is operationally defined as being resistant to high salt or detergents. The two components isolated during NM preparation are the internal NM and the nuclear lamina (NL) [8]. The internal NM can be observed in vivo during oogenesis, where it supports the chromosomes in large germinal vesicles of the oocyte [9,10,11,12,13,14,15], but its presence in ordinary somatic cells is questionable [16,17]. NL, which lies beneath the inner nuclear membrane, is a relatively insoluble fibrous structure [18]. The major NL components are lamins. Lamins are a type V family of intermediate filament proteins and perform structural and regulatory functions [19]. Lamins are generally divided into A and B types [20]. Lamins undergo complex modifications in the carboxyl terminus, which are required for their incorporation and subsequent assembly into NL. Mutations in the *Lmna* gene lead to defects in filament assembly and cause a wide variety of diseases collectively referred to as laminopathies. The silent mutation G608G in *Lmna* leads to the formation of permanently farnesylated progerin and causes Hutchinson–Gilford progeria syndrome (HGPS) [21]. Progerin, a defective lamin A, is toxic for cells and it has been suggested that its toxicity is associated with the farnesylated residue [22].

A-type lamins also localize in the nuclear interior. Specific functions of the nucleoplasmic lamin pool are poorly understood [23]. It is assumed that A-type lamins participate in the maintenance of telomere homeostasis [24,25] and the proper distribution of telomeres within nuclear space [25,26,27]. Lamin A/C deficiency and mutations lead to the accumulation of telomeres toward the nuclear periphery during interphase [25,27,28]. Lamin A/C interacts with TRF2 to promote the physical association of telomeres with interstitial chromatin through looping and to stabilize chromosome-end structure [28].

**Figure 1:**
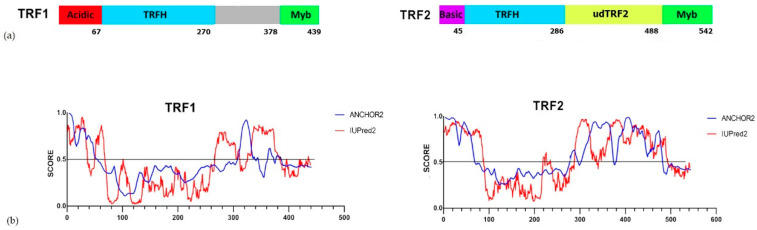
(**a**) Comparison of domain structures of TRF1 and TRF2. Basic, basic domain; Acidic, acidic domain; TRFH, TRF homology domain; Myb, DNA-binding Myb-domain. Numerals indicate the number of amino acid residues. (**b**) Prediction of the secondary structure dynamics of TRF1 and TRF2. IUPred2 (red) and ANCHOR2 (blue) scores are shown. The X-axis represents the amino acid number indicating its position in the sequence; the Y-axis represents the IUPred2 (red) scores that characterize the disordered tendency at each indicated position along the sequence; residues with a predicted score above 0.5 are considered disordered; ANCHOR2 (blue) scores characterize the probability of each residue being part of a binding region. The udTRF2 region of TRF2 is the intrinsically disordered region (IDR) that separates ordered TRFH and Myb domains. TRF1 also has an IDR between TRFH and Myb domains, but it is about half of udTRF2 and contains fewer residues, that could potentially be part of a binding region.

TRF1 and TRF2 have a similar domain structure [29] (Figure 1a). Between their DNA-binding Myb and homodimerization TRFH domains, both TRF1 and TRF2 have poorly conserved intrinsically disordered regions (IDRs) (Figure 1b). IDRs actively participate in diverse functions mediated by proteins, enabling the interaction of the same protein with a large number of partners [30,31,32]. In this study, the IDR of TRF2 is referred to as udTRF2. The amino acid sequence of udTRF2 is more variable among species than that of other TRF2 domains, though the dynamics of the secondary structure of the udTRF2 region are highly conserved (Figure S1).

UdTRF2 likely serves as an interface for interaction with different proteins. It would have been surprising if about one-third of the protein’s primary sequence had the sole function of connecting globular functional domains. We hypothesized that udTRF2 might be responsible for the interaction between TRF2 and lamins [1].

The recombinant protein corresponding to udTRF2 and polyclonal antibodies against this region were produced [33]. In the current work, the interaction between the udTRF2 recombinant protein and lamins from the NL extract was traced by co-immunoprecipitation. Mouse liver cell nuclei were used as the source of biological material. The interaction between recombinant udTRF2 and nucleoplasmic lamin A/C was confirmed by mass spectrometry (LC–MALDI). We found that udTRF2 is important for the interaction of telomere with lamins and that this interaction does not depend on post-translation modification nor require higher-order assembly of lamins. We also found an interaction between udTRF2 and some nuclear proteins. UdTRF2 may serve as an interface for protein–protein interactions that may play an important role in facilitating the functions carried out by the telomere complex.

## 2. Results

### 2.1. Antibodies against udTRF2

The recombinant protein corresponding to udTRF2 was expressed and purified and used to raise polyclonal antibodies (ABs) in guinea pig [33].

**Figure 2:**
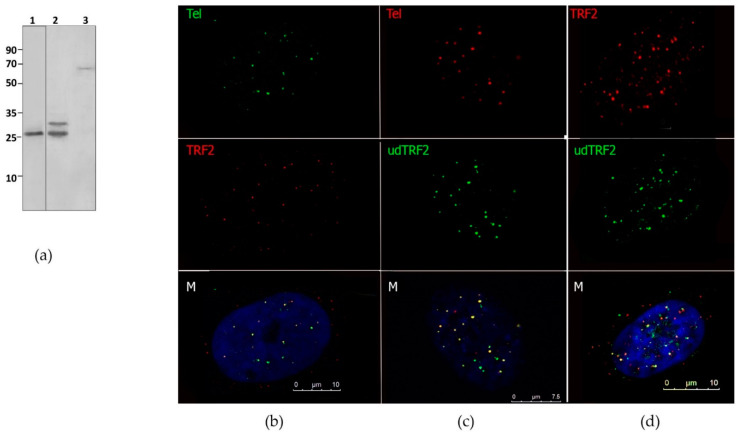
Antibodies (AB) against recombinant protein udTRF2 (the long linker region of TRF2). (**a**) Western blot of 1—induced bacteria lysate with commercial AB to TRF2 (Abcam); 2—induced bacteria lysate with anti-udTRF2 AB; 3—anti-udTRF2. AB reveals TRF2 in the human skin fibroblast lysate. Numbers on the left correspond to Mr of the marker. (**b**) ImmunoFISH staining of human skin fibroblasts: telomere probe—green; AB against TRF2 (ab13579, Abcam)—red. Scale bar = 10 µm. (**c**) ImmunoFISH staining of human skin fibroblasts: telomere probe—red; AB against udTRF2—green. (**d**) Immunostaining of human skin fibroblasts: AB against udTRF2—green; against TRF2 (ab13579, Abcam)—red. Scale bar = 7,5 µm. M—merged image. Representative confocal images, single z-slices, are shown. The results only indicate the coexistence of two molecules and do not provide any quantification.

In the epitope used to produce the commercial AB (ab4082, Abcam), there is a sequence that partly overlapped with udTRF2. We performed Western blotting of our anti-udTRF2 AB and the commercial AB under the same conditions to compare their specificities. The results of Western blotting showed that the commercial AB recognized a 25 kDa protein corresponding to udTRF2 in the induced bacteria lysate. Anti-udTRF2 AB recognized two proteins with Mr of 25 kDa (udTRF2) and 30 kDa in the induced bacterial culture lysate and a 70 kDa protein (TRF2) in human skin fibroblast lysate (Figure 2a) [33].

Immunofluorescence staining combined with in situ telomere hybridization (immunoFISH) with the telomeric probe and the commercial AB against full-length TRF2 (ab13579, Abcam) showed signal co-presence (Figure 2b). The ability of the polyclonal AB against udTRF2 to bind endogenous TRF2 in cells was tested on human fibroblasts using the same method (immunoFISH) (Figure 2c). We observed a marked coexistence between the signal from the telomere probe and the signal from udTRF2 AB. The signal shifts sometimes occurred with the AB against both full-length TRF2 and udTRF2 (Figure 2b,c). TRF2 is known to localize at internal telomere sequences, including the subtelomeric regions, to form secondary loop-like structures [28,34]. Double immunofluorescence staining was performed for both anti-udTRF2 and the commercial AB. The large foci of signals overlapping are visible on the merged image (Figure 2d). Slight differences in the staining between the two AB exist. The differences could be due to the nature of the AB; the commercial and udTRF2 ABs were produced against different parts of the TRF2 that only partially overlapped.

Double immunofluorescence staining was performed for both anti-udTRF2 and the commercial AB. We observed large foci corresponding to the signals overlapping on the merged image, and similar results were observed for the entire stained image (Figure 2d). Slight differences in the staining between the two antibodies were observed. The differences could be due to the nature of the AB; the commercial and udTRF2 ABs were produced against different parts of the TRF2 that only partially overlapped.

The results of Western blotting (Figure 2a) together with immunofluorescence and immunoFISH (Figure 2b–d) led us to conclude that the recombinant udTRF2 protein and corresponding polyclonal AB against it are suitable for use in co-immunoprecipitation.

### 2.2. udTRF2 Interacts with Lamins from Nuclear Lamina Extract

**Figure 3:**
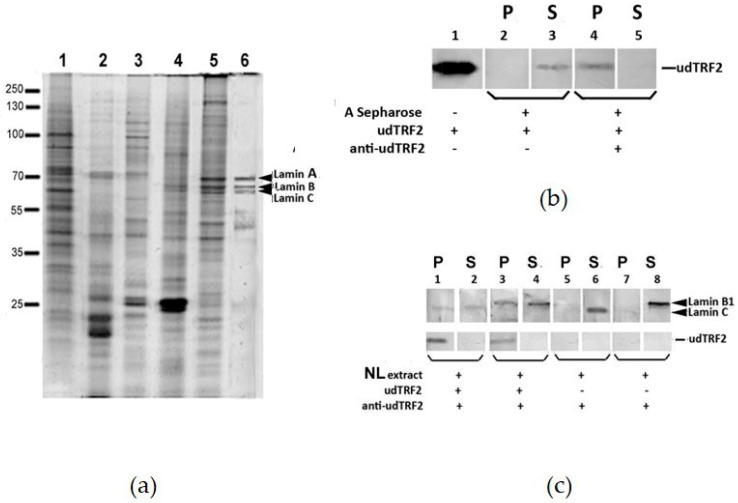
(**a**) Nuclear lamina (NL) purification and extraction (12% SDS-PAGE, CBB). Lane 1, soluble fraction after Triton; lane 2, soluble fraction after nuclease; lane 3, soluble fraction after LS buffer; lane 4, soluble fraction after HS buffer; lane 5, nuclear matrix (NM) precipitate; lane 6, NL extract with lamins A, B, C marked with arrows. Mr of marker indicated on the left. (**b**) udTRF2 AB evaluation. Lane 1, recombinant udTRF2 protein loaded; lane 2, control sample without anti-udTRF2 AB, Sepharose eluate; binding absent; lane 3, sample without anti-udTRF2 antibodies, supernatant, binding absent; lane 4, sample with anti-udTRF2 antibodies, Sepharose eluate, binding present; lane 5, sample with anti-udTRF2 antibodies, supernatant, binding present. P and S, pellet and supernatant from protein A Sepharose column, respectively. The input for each experiment is shown underneath and underlined with brackets. Anti-TRF2 AB (ab4182, Abcam) was used for Western blotting. (**c**) CoIP (co-immunoprecipitation) results analyzed with anti-lamin C and B1 ABs in the upper row and with TRF2 AB in the lower row. P and S, pellet and supernatant from protein A Sepharose column, respectively. The input for each experiment is shown underneath the brackets. Lamin positions are marked by arrows; udTRF2 position is marked by a dash.

The technique of lamina preparation and extraction can obtain the purest possible NL in the soluble state. Polypeptide profiles on SDS-PAGE showed that the final extract was enriched with lamins (Figure 3a). Three bands with Mr of 74, 68, and 63 kDa corresponding to lamins A, B, and C were clearly visible in the insoluble fraction and in the lamina extract (Figure 3a, lanes 5 and 6, arrows). The insoluble fraction also contained other proteins, which were apparently components of the nuclear pore complexes and other proteins, associated with NL; the NL extract contained much less contamination and nearly pure lamins.

AB that exhibits appropriate specificity may be ineffective as reagents for immunoprecipitation (IP). We assessed udTRF2 AB in IP with recombinant protein udTRF2 followed by Western blotting. Commercial rabbit polyclonal AB against TRF2 were used to check for udTRF2 binding. Western blotting showed that udTRF2 remained in the supernatant in the control sample without AB (Figure 3b, lanes 2 and 3) but was immobilized onto Sepharose in the sample with AB (Figure 3b, lanes 4 and 5). Thus, udTRF2 AB binds the native udTRF2 protein in solution and is suitable for co-immunoprecipitation (CoIP).

It was reported that lamins A/C are involved in telomere stability [28]. Only a few studies have been conducted on B-type lamins. Lamin B and TRF2 co-localized in double AB labeling [6]. Hence, we used AB against lamin B1 in parallel with lamin C AB in Western blotting after CoIP.

In the CoIP of the NL extract with recombinant udTRF2, lamin B1 bound to udTRF2 protein immobilized onto Sepharose by the AB (Figure 3c, lanes 3 and 4), whereas in the control samples, it remained in the supernatant (Figure 3c, lanes 7 and 8). Lamin C (A-type) also bound to the udTRF2 protein (Figure 3c, lanes 1 and 2). Different types of lamins interact with each other in vivo and in vitro [35,36,37]. Hence, we could not determine the type of lamins involved in the interaction between udTRF2 and the NL extract. It is unclear whether B-type lamins interact directly with TRF2 or through A-type lamins, but the CoIP results showed that a specific interaction occurs between the udTRF2 region and lamins.

### 2.3. udTRF2 Interacts with Soluble Lamin A/C from the Nucleoplasm

**Figure 4:**
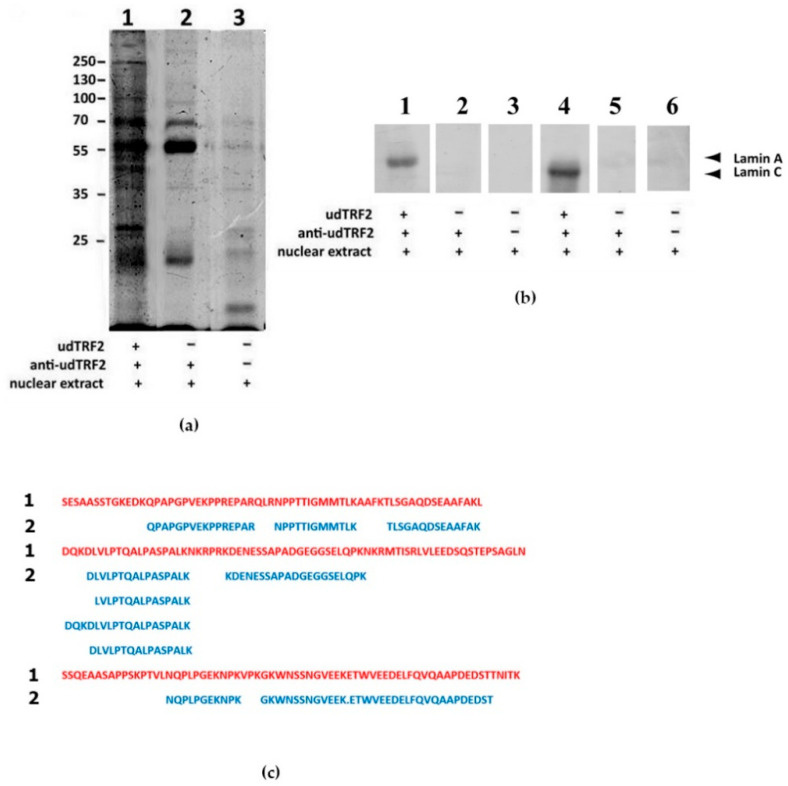
(**a**) Co-immunoprecipitation (12% SDS-PAGE, CBB). Lane 1, A Sepharose beads from a column loaded with udTRF2 AB, udTRF2, and nuclear extract (experiment); lane 2, column loaded with anti-udTRF2 AB only and nuclear extract (negative control 1); lane 3, A Sepharose beads from the column loaded with nuclear extract without AB (negative control 2). Mr of marker indicated on the left. (**b**) Western blot of A Sepharose columns. Lanes 1 and 4, A Sepharose beads from the column loaded with udTRF2 AB, udTRF2, and nuclear extract (experiment); lanes 2 and 5, column loaded with anti-udTRF2 AB only and nuclear extract (negative control 1); lanes 3 and 6, A Sepharose beads from the column loaded with nuclear extract without AB (negative control 2). Identical strips probed by lamin A and C AB in the upper row. Lamin positions are marked by arrows. Data are representative of three independent experiments using two independent nuclear extracts; (**c**) 1, udTRF2 sequence (red); 2, the peptide sequences found in LC–MALDI spectra (blue). All peptides defined as TRF2 belong only to the udTRF2 region.

Next, we considered the whole nuclear extract taking advantage of the use of soluble proteins. In an attempt to isolate udTRF2-interacting proteins, we performed CoIP using udTRF2 protein immobilized on protein A Sepharose using our developed AB as the bait for nuclear proteins. Two controls were used: in the first control, only anti-udTRF2 primary ABs were loaded on the protein -A column; in the second control, protein A Sepharose beads without AB were loaded. Proteins that remained on A Sepharose beads in the experimental sample were considered to be bound by udTRF2. SDS-PAGE revealed a complex mixture of udTRF2-interacting proteins that differed from those of the negative controls (Figure 4a). Hence, CoIP is successfully enriched by a unique set of proteins interacting with udTRF2. Both lamins of A type (A and C) bound to the udTRF2 (Figure 4b).

CoIP followed by LC–MALDI analysis were used to identify proteins interacting with udTRF2. All peptides from the experimental sample defined as TRF2 belonged only to the udTRF2 linker region (Figure 4c); peptides corresponding to other TRF2 regions were absent. Hence, all the other proteins in the sample interacted with this region, though not necessarily directly. Among the peptides in the experiment sample, there were peptides encoded by *Lmna* in two of three biological replicates, as expected (Table 1). All identified peptides belong to lamins A and C or immature prelamin A/C (Supplementary Table S1). Peptides encoded by *Lmna* were not identified in control samples. The interaction of udTRF2 with lamins A and C, as revealed by Western blot (Figure 4b), was confirmed by mass spectrometry. Remarkably, lamin-associated proteins were absent among udTRF2-associated proteins, demonstrating that the interaction between lamin A/C and TRF2 was not dependent on such proteins.

Thus, we concluded that the udTRF2 region can mediate interactions between lamin A/C and TRF2.

### 2.4. Non-Lamin Interacting Partners of udTRF2

Other udTRF2-interacting proteins in the CoIP sample were identified using LC–MALDI (Table 1). These proteins were not present in the control samples.

Represented in the udTRF2 interactome were several heterogeneous nuclear ribonucleoproteins (hnRNP A2/B1, hnRNP A1, hnRNP A3, hnRNP K, hnRNP L, and hnRNP M), splicing factors (SFPQ, NONO, SRSF1, and others), helicases (DDX5, DHX9, and Eif4a3l1), topoisomerase I, and heat shock protein 71 (Table 1). DNase/RNase I digestion was applied during extraction to avoid unspecific reactions with nucleic acids. Still, some of the identified proteins are known to bind single-stranded telomeric DNA/RNA [38]. Nucleic acid depletion presupposes that direct protein–protein interactions occur. A set of proteins involved in the regulation of DNA and RNA secondary structures, such as G-quadruplexes and R-loop, was identified.

HnRNPs are a large family of proteins. Members of the hnRNP family are involved in pre-mRNA processing and mRNA export [39,40], and are implicated in telomere maintenance. For example, hnRNP A2/B1 protects the telomeric DNA repeat region from endonuclease digestion [41]. Some members of the hnRNP family have been reported to be part of a telomerase complex that negatively regulates telomere length [42,43]. Hence, hnRNPs, which bind both single-stranded RNA and DNA, could control the accessibility of telomeric 3′ overhangs [44]. Telomere extension depends on the conformation of the telomeric single-stranded 3′ overhangs, and their folding into secondary structures, known as G-quadruplexes, prevents the extension of telomeres by telomerase [45]. Telomere repeat-containing RNA (TERRA) is also prone to forming RNA:DNA hybrids with C-rich telomeric strands, producing R-loop structures [46]. R-loop levels are strictly regulated due to the potential threats they pose. Several reports have stated that helicases are involved in their unfolding [47,48]. SFPQ and NONO suppress R-loop formation and play a common role in suppressing replication defects at telomeres, telomere fragility, and telomere recombination [49]. They have been reported to be associated with TRF2 [49]. Future studies should determine the functional implications of the interactions between udTRF2 and the remaining co-isolated proteins.

## 3. Discussion

### 3.1. Interaction between TRF2 and Lamins

In mammals, most telomeres are distributed throughout the nuclear volume during interphase [50]. Defects in their distribution are associated with age-related diseases, including cancer, in addition to several premature aging syndromes. Despite the importance of this phenomenon, the molecular mechanisms of telomere distribution in mammalian cells remain obscure. It is thought that telomere distribution in the nuclear interior is mediated by lamins A/C, which act as mechanical linkages. The interaction between TRF2 and soluble lamin A/C from nuclear extract has been reported [28]. The lamin A–TRF2 association seems to be important not only for telomere stability [28] but also for telomere localization within the nucleus. Although the majority of mammalian telomeres are distributed in the nuclear interior, some telomeres have nuclear peripheral localization [51,52,53]. Subtelomeric sequences, such as heterochromatic sequences, could drive an individual telomere to the NL [54]. This type of interaction does not exclude the mechanism via TRF2. It has been shown that progerin (defective lamin A) is unable to interact properly with TRF2 [28]. The progerin-like mutations of A-type lamins result in various alterations in telomere structure and function, such as impaired maintenance of telomere-length homeostasis and changes in the spatial distribution of telomeres [24,25,26,27,28,55,56].

It was previously shown that GFP-TRF2ΔBΔM, a mutant of TRF2 that lacks the basic N-terminal and DNA-binding domains but contains udTRF2, was not able to interact with lamin A/C [28]. In contrast to this study, we found that udTRF2 interacts with nucleoplasmic lamin A/C. It is possible that the function of the native protein was altered by the GFP tag. GFP and its derivatives are widely used in vitro and in vivo, and the use of GFP fusion protein is considered to have negligible effects on cellular function. However, a number of reports have shown that GFP tagging may impact the biological activity of proteins due to conformational changes [57,58,59]. The basic N-terminal domain of TRF2 is flexible (Figure 1 and Figure S1) and the negative effects of GFP tagging may be mitigated by inserting a linker at the fusion point [59]. Hence, the conformational changes arising from GFP tagging could significantly reduce this interaction. Only a small fraction of the total TRF2 and lamin A/C interacts [28], and some research groups did not detect an interaction between TRF2 and lamin A [60]. Our approach visibly shows evidence of an interaction between udTRF2 and lamin A/C.

It was assumed that lamin A/C only interacts with functional DNA-bound TRF2 [28]. This conclusion is based on the lack of interaction between TRF2ΔBΔM and lamin A/C. TRF2 undergoes arginine methylation [61], and methylated TRF2 is largely not localized at telomeres, but lamin A staining was observed to overlap with methylated TRF2 staining at the nuclear periphery of senescent cells [60]. Our data also indicate that the interaction between TRF2 and lamins is likely to be independent of telomere DNA.

Lamin A/C could mediate nuclear envelope–telomere attraction. TRF2 co-localized with lamins in the nuclear envelope of the oocyte nucleus of *Rana temporaria* [13]. We demonstrated that udTRF2 interacted with purified lamins from the NL extract. We did not use the insoluble higher-order NL fibrous structure though it is highly probable that lamins from the NL extract exist in a mature, post-translationally modified state. The finding that udTRF2 interacts with both nucleoplasmic lamins and lamins from NL indicates that the interaction does not depend on post-translational modifications of lamins. The lamins of both types bound to udTRF2 in CoIP experiments (Figure 3). It is unclear whether B-type lamins interact with TRF2. In mammalian cells, a larger number of telomeres has been observed near the NL immediately after mitosis [62]. B-type lamins remain associated with the membrane throughout the cell cycle, whereas A-type lamins accumulate in the nuclear interior in early prophase and assemble into the lamina throughout telophase [63,64,65,66,67,68]. It seems that a low content of lamin A/C in the NL is not sufficient for supporting telomere attachment in early telophase and that other partners are necessary for binding. TRF2 colocalization with lamin B in nuclear envelope remnants has been observed in metaphase mouse cells [6]. Thus, it cannot be ruled out that B-type lamins could be the TRF2 binding partner. However, we did not find any B-type lamins among the interacting partners in the mass spectrometry results (Table 1). The interaction between lamin A/C and udTRF2 seems more probable, but B-type lamins form stable structures and are largely insoluble [23,63]. Different types of lamins interact with each other in NL. The possibility of lamin B–TRF2 interaction should be examined using other methods.

### 3.2. Interaction between udTRF2 and Other Proteins

A set of proteins involved in the interaction with udTRF2, including hnRNPs, helicases, splicing factors, and others, was identified by mass spectrometry (Table 1). TRF2 is known to exist in association with the nuclear matrix (NM) [69,70,71,72]. Telomeres attach to the NM [3,4]. Consequently, integral components of the NM, such as hnRNPs [73] and members of the DExD/H family [74], were identified among the udTRF2-binding proteins. These proteins are potential candidates for facilitating telomere association with the NM through TRF2. Our experiments indicate that the udTRF2 region is the key point for the association of TRF2 with the NM.

A set of proteins involved in the regulation of DNA and RNA secondary structures such as G-quadruplexes and R-loops was identified among the udTRF2-interacting proteins. Some of them are known components of telomeric chromatin. Members of the hnRNPs family can regulate the formation and activity of G-quadruplexes and play multiple roles pertaining to telomere DNA protection [44]. SFPQ/NONO foci co-localize with TRF2 [49], which is consistent with our data. Both NONO and SFPQ participate in telomere length homeostasis and suppress R-loop-related telomere fragility and recombination, although both proteins do not possess enzymatic activity [49]. However, a number of other proteins identified among the udTRF2-associated proteins could participate in this regulation.

G-quadruplex and R-loops at the telomeres or transcribed regions of the genome are regulated by G-quadruplex-binding proteins and play a role in various cellular functions including chromatin regulation, transcription, initiation of DNA replication, telomerase activity, and promoting homologous recombination among telomeres [46]. Many RNA and DNA helicases resolve RNA/DNA G-quadruplexes that would otherwise pose an obstacle to DNA replication. Topoisomerases TOP1 and TOP3B play a key role in alleviating topological strain during transcription, and their deficiency accumulates R-loops [75]. TRF2 binds potential G-quadruplex sequences within the telomere, regulates gene expression in some promoter regions of the genome [76], and, in addition, manages specific topological problems during telomeric replication [77,78,79]. It acts in pathways complementary to TOP2α, and perhaps TOP2β, to protect telomeres during replication [79]. Hence, the interaction between TRF2 and TOP1 is possible. The interaction of heat shock protein hsp70 with topoisomerase I protects topoisomerase activity from heat stress [80].

Future studies may help to reveal the functional significance of these interactions. The proteins newly found as TRF2 binding might shed light on obscure questions in telomere biology.

We found that the udTRF2 region is responsible for the TRF2–lamin interaction. The interactions between TRF2, lamins, and other nuclear proteins are mediated by udTRF2, the long linker region that is poorly conserved and intrinsically disordered but has highly conserved dynamics in its secondary structure. TRF2, and its region containing such features, appears to be the main player in the complex process of telomere positioning in the nucleus.

## 4. Materials and Methods

### 4.1. Protein Expression and Purification

A purified solution of recombinant udTRF2 protein was prepared as previously described [33].

### 4.2. SDS-PAGE and Western Blotting (WB)

SDS-PAGE separation was performed according to Laemmli [81]. The proteins were visualized by Coomassie Brilliant blue G250 (CBB) staining. After separation in SDS-PAGE, the proteins were transferred onto PVDF membranes (Thermo Fisher Scientific Rockford, IL, USA) following overnight incubation with specific primary antibodies. The following antibodies were used: TRF2–anti-TRF2 (ab4182, Abcam, Cambridge, UK, dilutions 1:1000), anti-udTRF2 ([33] dilutions 1:500); lamin C–anti-lamin C (ab97774, Abcam, Cambridge, UK, dilutions 1:500); lamin B1–anti-lamin B1 (ab231282, Abcam, Cambridge, UK, dilution 1:2000); lamin A–anti-lamin A (133a2, Abcam, Cambridge, UK, dilutions 1:500).

### 4.3. ImmunoFISH

A certified culture of human foreskin dermal fibroblasts was obtained from the Stem Cell Bank Pokrovsky (St. Petersburg, Russia). Briefly, human dermal fibroblasts were obtained from a foreskin sample of a 5-year-old donor after written informed consent was signed by his parents. This sample was thoroughly washed with 1× PBS (Life Technologies, Foster City, CA, USA) and minced into small pieces using a surgical scalpel prior to digestion with a 0.1 mg/mL mix of collagenase type I and IV (Thermo Fisher Scientific, Waltham, MA, USA) in 1× PBS on a shaker platform at 37 °C for 1 h. Then, the solution was transferred into a 15 mL tube and centrifuged. The pellet was digested again with 0.1 mg/mL mix of collagenase type I and IV/0.25% trypsin (1:1) in 1× PBS and incubated again in the same conditions. After centrifugation at 400 g for 7 min, the pellet was resuspended in a Dulbecco’s modified Eagle’s low glucose medium (DMEM LG GlutaMAX, Gibco, Gaithers, MD, USA) supplemented with 10% FBS (fetal bovine serum; HyClone, Salt Lake City, UT, USA), 100 U/mL penicillin, and 100 μg/mL streptomycin (Gibco, Gaithers, MD, USA). The cells were seeded into a 25 cm^2^ T-flask (TPP, Trasadingen, Switzerland) and cultured at 37 °C in DMEM with 4.5 g/L glucose supplemented with 10% bovine fetal serum and antibiotic/antimycotic (penicillin/streptomycin) mixture at 5% CO_2_. At passage 5, cells were seeded onto coverslips and cultured until the confluency reached 60–70%. The coverslips with cells were then washed with 1× PBS and fixed either in methanol/glacial acetic acid or in 4% paraformaldehyde fixatives. Washed and fixed cells were incubated in antibody solutions (anti-udTRF2, dilutions 1:200; anti-TRF2 ab13579, Abcam, Cambridge, UK) for 16 h at 4 °C and then incubated in a solution of secondary antibodies (goat antibodies against guinea pig immunoglobulins conjugated to Alexa-488, dilutions 1:200, A11073, Invitrogen, Carlsbad, CA, USA; donkey antibodies against mouse immunoglobulins conjugated to Alexa-568, dilutions 1:200, A11037, Invitrogen, Carlsbad, CA, USA) for 1 h at room temperature. Then, cells were additionally fixed, treated with RNase (0.1 mg/mL, Thermo Fisher Scientific, Waltham, MA, USA), and dehydrated in a series of increasing ethanol solution (70%, 90%, and 96% ethanol). For hybridization, we used a double-stranded telomeric probe size 300–1000 bp labeled with biotin (DNA Synthesis, Moscow, Russia). Probe and drug were denatured in 70% formamide on 2× SSC with 10% dextran sulfate at 80 °C for 5 min and then hybridized at 37 °C for 16 h. The preparations were washed in 45% formamide 2× SSC at 42 °C for 5 min, and then twice in 2× SSC for 5 min, and incubated in a solution of streptavidin conjugated with Alexa-568 or Alexa-488 (Thermo Fisher Scientific, Waltham, MA, USA) at a dilution of 1:200 for 1 h. The preparations were mounted in Antifade Gold with DAPI (Thermo Fisher Scientific, Waltham, MA, USA).

### 4.4. Confocal Microscopy

The preparations were analyzed with a Scanning Confocal Microscope Leica TCS SP5 (Leica Microsystems, Wetzlar, Germany) equipped with a 63× oil objective (HCX PL APO, lambda blue) with a numerical aperture (N.A.) = 1.4. For image acquisition, we used UV (405), argon (488 nm), and helium–neon (543 nm) laser sets. To avoid crosstalk between fluorophores, a sequential scan was performed. Co-presence events were microscopically evaluated based on merged yellow signals in cell nuclei.

### 4.5. Nuclear Proteins Fractionation

Nuclei were isolated from adult mouse livers and nuclear fractionation was performed according to Kaufmann [82]. Briefly, nuclei were incubated in STM buffer (50 mM Tris, pH 7.5, 5 mM MgSO_4_, 0.25 M sucrose, 0.5 mm PMSF) with 1% Triton X-100 for 10 min at 0 °C and after centrifuged for 10 min at 1500 g. The precipitate was dissolved in STM buffer with 0.1 mg/mL DNase (Thermo Fisher Scientific, Waltham, MA, USA) and 0.1 mg/mL RNase, incubated for 30 min at room temperature, and centrifuged for 10 min at 1500× *g*. The precipitate was dissolved in LS buffer (10 mM Tris, pH 7.5, 5 mM MgCl_2_, 0.5 mm PMSF), incubated for 15 min at 0 °C, centrifuged for 10 min at 1500× *g*. The precipitate was dissolved in HS buffer (LS buffer with 1.6 M NaCl), incubated for 15 min at 0 °C, and centrifuged for 10 min at 1500× *g*. Supernatants were analyzed using SDS-PAGE separation. Insoluble precipitate of the nuclear matrix was dissolved in buffer (30 mM Tris, pH 9.0, 1% Triton X-100, 0.5% 2-mercaptoethanol, 0.5 mm PMSF) for 8 h at 4 °C; insoluble proteins were pelleted by centrifugation for 10 min at 10,000× *g*; the supernatant was concentrated using Vivaspin 500 filters (Sartorius Stedim Biotech, Aubagne, France). The fraction containing a mixture of lamins was used for CoIP. Nuclear extracts were prepared from mouse liver nuclei following the method of Abmayr et al. [83].

### 4.6. Immunoprecipitation (IP) and Co-Immunoprecipitation (CoIP)

IP and CoIP were performed according to recommended procedures (www.invitrogen.com/content.cfm?pageid=10678, accessed on 1 February 2018). Briefly, anti-udTRF2 antibodies were incubated with protein A Sepharose (101042, Thermo Fisher Scientific, Waltham, MA, USA) for 3 h in immunoprecipitation (IP) buffer (20 mm Tris pH 7.5, 150 mm NaCl, 1 mm EDTA, 1 mm PMSF). Then, 5 µL of purified recombinant protein udTRF2 solution (concentration 250 µg/mL) was immunoprecipitated with anti-udTRF2 polyclonal antibodies bound to protein A Sepharose for 7 h and washed three times in IP buffer. All procedures were performed at 4 °C.

For CoIP, nuclear lamina extracts were incubated with 5 μL udTRF2 solution (0.25 mg/mL) for 7 h, and was then added to protein A Sepharose with the appropriate antibodies. As a control, protein A Sepharose was incubated with the lamina extracts; protein A Sepharose with antibodies was incubated with the anti-udTRF2 and with lamina extract. To identify proteins capable of interacting with the udTRF2 linker region, antibodies were immobilized in protein A Sepharose was incubated with an excess of recombinant udTRF2 protein (5 mg) for 3 h and then the nuclear extract was added. To control nonspecific binding, the nuclear extract was incubated with anti-udTRF2 antibodies immobilized in protein A Sepharose under similar conditions as indicated in Figure 4.

### 4.7. Mass Spectrometry and Protein Identification

Proteins from CoIP probes were denatured in 8 M urea and sequentially treated with dithiothreitol and iodoacetamide. The reaction mixture was diluted 10 times by adding 50 mM Tris-HCl, pH 8.0. A total of 1 μg of trypsin (Trypsin Gold, V5280, Promega, San Luis Obispo, CA, USA) was then added to the solution. The reaction was carried out by incubation overnight at 37 °C. Peptides were isolated by solid-phase extraction on reverse-phase cartridges (30 mg, Strata-X, 8B-S100-TAK, Phenomenex, Aschaffenburg, Germany) and dried in a Martin Christ RVC 2-33 IR rotary vacuum concentrator (Martin Christ, Osterode am Harz, Germany). The resulting peptide mixture was separated on a 50 mm × 1 mm reversed-phase column (BioBasic C18, Thermo Scientific, Waltham, MA, USA) using a water/acetonitrile gradient on a Milichrom A-02 microcolumn HPLC system (Ekonova, Novosibirsk, Russia). Eluates were fractionated and applied to MALDI targets using a robotic microfraction collector. Mass spectrometric studies were conducted on an AB Sciex TOF/TOF 5800 mass spectrometer (AB Sciex, Redwood City, CA, USA) in the reflectron (MS) and tandem mass spectrometry (MS/MS) modes. Fragment ion MS/MS spectra were searched using the MASCOT search tool against the UniProtKB/Swiss-Prot protein database using appropriate parameters. Mass spectra were acquired using TOF/TOF Series Explorer software (AB Sciex, Redwood City, CA, USA). MS and MS/MS spectra were analyzed using the specialized software ProteinPilot 4.0 (AB Sciex, Redwood City, CA, USA) according to the UniProtKB international protein database.

### 4.8. Bioinformatics Analysis

To predict the dynamics of the secondary structure of the TRF2 and TRF1, the IUPred predictor (http://iupred.enzim.hu accessed on 15 August 2019) was used.

## Figures and Tables

**Table 1:** Proteins identified in the udTRF2 interactome.

Gene Name	UniProt ID	Protein Name	Number of Peptides (95%)
*LMNA*	P48678	Prelamin-A/C	4
*Hnrpa2b1*	O88569	Heterogeneous nuclear ribonucleoprotein A2/B1	16
*ROA3*	Q8BG05	Heterogeneous nuclear ribonucleoprotein A3	10
*TADBP*	Q921F2	TAR DNA-binding protein 43	4
*HNRPK*	P61979	Heterogeneous nuclear ribonucleoprotein K	3
*HNRPM*	Q9D0E1	Heterogeneous nuclear ribonucleoprotein M	2
*ROA1*	P49312	Heterogeneous nuclear ribonucleoprotein A1	2
*HNRPL*	Q8R081	Heterogeneous nuclear ribonucleoprotein L	1
*SFPQ*	Q8VIJ6	Splicing factor, proline- and glutamine-rich	5
*NONO*	Q99K48	Non-POU domain-containing octamer-binding protein	3
*SRSF1*	Q6PDM2	Serine/arginine-rich splicing factor 1	3
*FBRL*	P35550	rRNA 2′-O-methyltransferase fibrillarin	3
*U520*	Q6P4T2	Small nuclear ribonucleoprotein U5 subunit 200	3
*SMD3*	P62320	Small nuclear ribonucleoprotein Sm D3	1
*DKC1*	Q9ESX5	H/ACA ribonucleoprotein complex subunit DKC1	1
*SMU1*	Q3UKJ7	WD40 repeat-containing protein SMU1	1
*PRP19*	Q99KP6	Pre-mRNA processing factor 19	1
*PHF5A*	P83870	PHD finger-like domain-containing protein 5A	1
*PRP8*	Q99PV0	Pre-mRNA-processing-splicing factor 8	1
*H0V9E4*	H0V9E4	Probable ATP-dependent RNA helicase DDX5	4
*DHX9*	O70133	ATP-dependent RNA helicase A (DHX9)	7
*E9PV04*	E9PV04	Eukaryotic translation initiation factor 4A3-like 1	1
*TOP1*	Q04750	DNA topoisomerase I	2
*HSP7C*	P63017	Heat shock cognate 71 kDa protein	3

## Supplementary Materials

Supplementary materials can be found at https://www.mdpi.com/1422-0067/22/7/3293/s1.

## Abbreviations

AB	antibodies
CBB	Coomassie brilliant blue
CoIP	co-immunoprecipitation
IP	immunoprecipitation
LAP2α NL	lamin-associated protein 2α nuclear lamina
NM	nuclear matrix
Mr	apparent molecular weight
