# Supplementary material for: The Long Linker Region of Telomere-Binding Protein TRF2 Is Responsible for Interactions with Lamins
Source: Int J Mol Sci. 2021 Mar 24;22(7):3293. doi: 10.3390/ijms22073293 (PMC8036907; doi:10.3390/ijms22073293)
Supplement: Supplementary file 1 [file ijms-22-03293-s001.pdf]

Figure S1. Prediction of secondary structure dynamics of TRF2 using the IUPred predictor (http://iupred.enzim.hu). IUPred2 scores characterize the disordered tendency of each position along the sequence, residues with a predicted score above 0.5 are considered disordered; ANCHOR2 scores characterize the probability of each residue being part of a binding region.

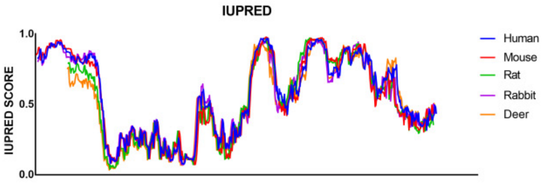

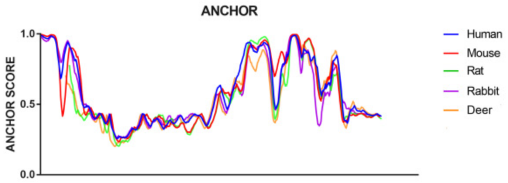

Table S1. Mass spectrometric analysis reveals that peptides are common to lamins A and C or immature prelamin A/C. Peptides in the first column were detected at least once in two independent runs.

Peptide	Residues in prelamin A/C
LQEKEDLQELNDR	29–41
LQTLKEELDFQK	198–210
LADALQELR	241–250
NSNLVGAAHEELQQSR	280–296
VAVEEVDEEGKFVR	439–453
AQNTWGCGSSLR	516–529
